# Supplementary material for: Urban Neighbourhood Environments, Cardiometabolic Health and Cognitive Function: A National Cross-Sectional Study of Middle-Aged and Older Adults in Australia
Source: Toxics. 2022 Jan 7;10(1):23. doi: 10.3390/toxics10010023 (PMC8779212; doi:10.3390/toxics10010023)
Supplement: Supplementary file 1 [file toxics-10-00023-s001.zip › toxics-1521015-supplementary-for proof 1.6.pdf]

toxics

MDPI

Supplementary Materials: Urban Neighbourhood Environments, Cardiometabolic Health and Cognitive Function: A National Cross-Sectional Study of Middle-Aged and Older Adults in Australia

Authors: Ester Cerin, Anthony Barnett, Jonathan E. Shaw, Erika Martino, Luke D. Knibbs, Rachel Tham, Amanda J. Wheeler and Kaarin J. Anstey

Section S1. Detailed Description of Built and Natural Environmental Exposures

Measures of the neighbourhood built and natural environment were generated using ESRI's ArcGIS v.10.5 software (ESRI, Redlands). Participants' residential addresses were geocoded and 1-km untrimmed street-network buffers were created around the geocoded locations following procedures employed in international studies of neighbourhood environmental determinants of health-related behaviours and obesity in adults and older adults [1–3]. A 1–km radius was used to create residential buffers because it corresponds to the distance that adults and older adults without mobility problems can cover in a 10-20 minute walk [1], and the latter is commonly used to define a neighbourhood [4,5].

Four built environment measures were computed for each participant's residential buffer. These included population density, street intersection density, percentage of commercial land use and an entropy score denoting the heterogeneity of non-commercial land use. Population density, here defined as the number of persons per hectare, was derived using the Australian Bureau of Statistics (ABS) Mesh Block data from the 2011 Census [6]. Mesh Blocks are the smallest geographical areas defined by the ABS for which Census data are available. Population density was selected as an environmental exposure of interest because it is the primary driver of changes in the built environment [7,8] and is consistently linked to active transport [9]. It may also facilitate social activities and confer associated cognitive benefits [10]. Street intersection density, defined as the number of ≥3-way intersections per km^2, was computed using road network data derived from the PSMA Australia's 2012 Transport & Topography dataset [11]. As this neighbourhood feature provides easier access to public transport and destinations, it is deemed to facilitate walking and engagement in cognition-enhancing activities [7]. However, it may also result in greater personal exposure to air pollution in trafficked areas and, hence, harm residents' well-being [12]. The percentage of buffer area devoted to commercial land use was derived from 2011 ABS data on the main planned land use for Mesh Blocks [13]. Commercial land uses that support good service provision, such as shops and supermarkets provide destinations for various activities [8,9]. A land use entropy score or land use mix ranging from 0 to 1 [14] and denoting the heterogeneity of non-commercial five land use categories (i.e., residential, industrial, medical, educational and other land uses from 2011 ABS Mesh Block data)[13] was computed to quantify accessibility of various non-commercial destinations that may promote active transport and engagement in activities [7,15].

Two natural environment measures were included in this study: percentage of residential buffer area covered by parkland derived from 2011 ABS Mesh Block data [13] and percentage of buffer area cover by waterbodies or blue spaces (e.g., lakes, coastlines, rivers and reservoirs) derived from national topographic spatial data for surface water features sourced from Geoscience Australia [16]. Access to parks has been relatively consistently associated with higher levels of physical activity [8,9], better mental health [17], more social contacts [18] and, in some studies, better cognitive function [19]. Green spaces also mitigate ambient air pollution [20]. Access to fresh water and navigable waterbodies are

of crucial importance to humans [21,22]. A few studies have also found blue space accessibility to promote physical activity [23] and better mental health [24] due to its restorative properties (e.g., stress reduction) [25] and its role in promoting social interactions [26].

Section S2. Detailed Description of Analytical Steps

Descriptive statistics and percentage of missing values were computed for all variables. Over 17% of cases had missing data on at least one variable and 4.5% on more than three variables. Predictors of missingness (the odds of having incomplete data on any of the examined variables) were determined using generalized linear mixed models with binomial variance and logit link functions and random intercepts at the Statistical Area 1 (SA1) level. The odds of having missing data were higher in older participants ($p < .001$), those of non-English speaking background ($p < .001$), with lower household income (p=.022), not working or volunteering ($p = .008$), living in areas with lower socio-economic status (p=.030) and with lower scores on the memory test ($p = .036$). Missingness was also more prevalent in people for whom access to services was an important reason for living in their neighbourhood ($p = .040$) and those living in areas with higher population density ($p < .001$), lower street intersection density ($p = .010$) and lower concentrations of $PM_{2.5}$ ($p < .001$). As data were at least missing at random (MAR) rather than missing completely at random (MCAR), ten imputed datasets were created for the regression analyses as recommended by Rubin [26] and van Buuren [27]. Multiple imputations by chained equations were performed following currently recommended model-building and diagnostic procedures [27] and using the package 'mice' [28] in R version 4.0.0 [29].

The aim of this study was to examine the extent to which the potential effects of neighbourhood environment characteristics on cognitive function are explained by cardiometabolic risk factors. Generalised additive mixed models (GAMMs; package 'mgcv' version 1.8.22 [30] in R) with random intercepts at the SA1 level were used for this purpose to account for curvilinear relationships of unknown form and spatially correlated data [30]. Here, the meaning of 'effect' needs to be interpreted in the context of the cross-sectional observational nature of the study with possible unmeasured confounders. Directed acyclic graphs (DAGs) were used to inform the selection of a minimal sufficient set of confounders to be included in the GAMMs estimating exposure-mediators and mediators-outcomes relationships (Figure S1). The DAGs were based on the hypothesised causal effects among the variables according to previous studies (see Introduction/Background and Methods sections in the paper and explanation below) and the authors' expert opinion. Potential multicollinearity was assessed by computing the Variance Inflation Factor (VIF) for each variable included in the GAMMs. All VIFs were smaller than 2.58, indicating no collinearity issues [31]. Analyses were conducted in several steps described below.

Mediated and direct effects of neighbourhood environmental characteristics on cognitive function

Mediation was examined using the joint-significance test [7,32] according to which data support mediation if the associations (regression coefficients) between an exposure and its mediator(s) and the exposure-adjusted associations between the mediator(s) and the outcome are both statistically significant ($p < .05$). This was done in several steps. Given that our analyses considered potential causal effects among environmental characteristics, we first estimated the confounder-adjusted total effects of each environmental variable on each cardiometabolic risk factor (Table S1). Here, 'total effect' refers to the sum of effects mediated and unmediated by other environmental variables and is estimated by excluding from the regression model those environmental characteristics that are deemed to be in the pathway between the environmental exposure of interest and the response variable (outcome or mediator) (steps 1Ta to 1Th; the letter 'T' refers to 'total effect'). GAMMs with Gaussian variance and identity link functions were used to model waist circumference, LDL cholesterol and mean arterial pressure, while Gamma variance and logarithmic link functions were used to model the remaining cardiometabolic risk factors. Curvilinear associations were estimated using smooth terms modelled with thin plate splines [30]. If the data did not provide sufficient evidence of a curvilinear association, smooth terms were

replaced by linear terms. Model selection (linear vs. curvilinear effect) was based on Akaike Information Criterion (AIC) values, where a lower AIC was indicative of a better-fitting model. A median ≥5-unit difference in AIC across all imputed datasets was used as the criterion for linear vs. curvilinear effect selection [33,34].

In step 2, we estimated the direct effects of specific environmental attributes on other environmental attributes. This entailed regressing street intersection density, percentage of commercial land and land use mix (5 non-commercial land uses) onto population density (step 2Da in Table S1; the letter 'D' refers to 'direct effect') for the reasons explained in the Introduction/Background of the paper. Environmental characteristics potentially influencing the percentage of parkland in residential buffers were examined in step 2Db and those of air pollutants were assessed in step 2Dc. Increases in population density above a certain threshold, and the resulting expansion of residential, commercial, industrial and similar land uses, were hypothesised to lead to a reduction in parkland [7]. Activities resulting from higher levels of population density, street intersection density, commercial land and similar land uses were hypothesised to yield an increase in air pollution [35], while the proportion of parkland was hypothesised to mitigate air pollution levels [20].

In step 3, we estimated the 'direct effects' of environmental characteristics on the cardiometabolic risk factors (i.e., unmediated by other environmental variables) by including in the regression models all environmental characteristics hypothesised to mediate the effects of the environmental exposure of interest on the response variable. As we hypothesised that adiposity (waist circumference) would be a determinant of other cardiometabolic risk factors [36,37], we also estimated the direct effects of waist circumference on other cardiometabolic risk factors adjusted for all environmental variables (steps 3Da to 3Df in Table S1). In this step, we also examined whether taking medications for a specific cardiometabolic risk factor moderated the associations of environmental characteristics, waist circumferences with the cardiometabolic risk factor.

Step 4 of the mediation analyses estimated the direct effects of environmental attributes and cardiometabolic risk factors on the two measures of cognitive function (step 4D in Table S1). Medications for diabetes, hypertension and dyslipidaemia were considered as potential moderators of specific direct or indirect effects of environmental factors on cognitive function. The impact of cardiometabolic risk factors and their environmental determinants on cognitive function may depend on whether a person is taking medications for cardiometabolic conditions [38].

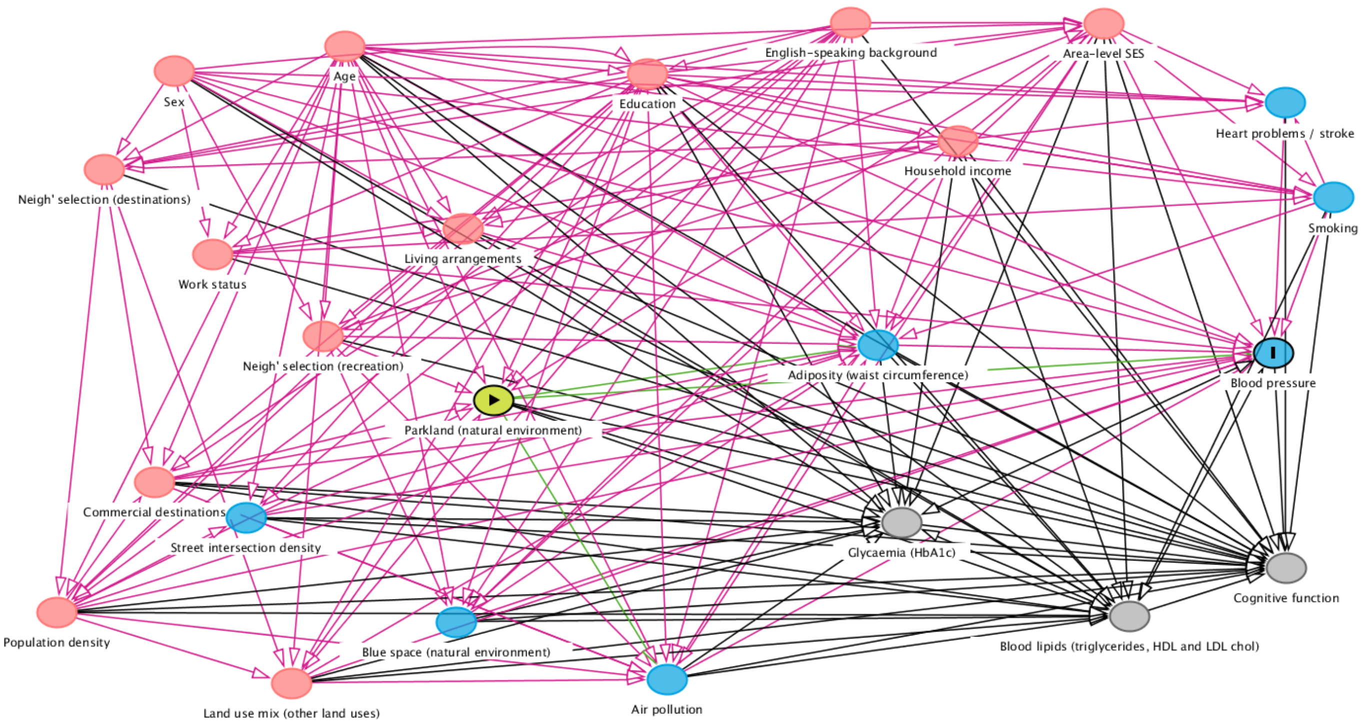

Figure S1. Directed acyclic graph (DAG) depicting the hypothesised relations between neighbourhood attributes, cardiometabolic risk factors and cognitive function. Through the DAG, we identified which covariates to include in the statistical analyses to sufficiently control for potential confounders. This particular DAG was used to inform the model of the total effect of percentage of parkland in the neighbourhood on mean arterial pressure (blood pressure). Variables with red circles denote the set of potential confounders. A minimal sufficient set of confounders (included in the regression models) is a subset of this set of variables.

Table S1. Outline of regression analyses.

Estimation of Total Effects of Neighbourhood Environmental Attributes on CardiometabolicR Factors			
**Step**	**Exposure(s) / effect(s)**	**Covariates**	**Regression models**
1Ta*	Population density (persons/hectare)	Age, sex, English-speaking background, living arrangements, educational attainment, area-level IRSAD, residential self-selection related to access to destinations	Six separate sets of GAMMs (one GAMM with a linear and another with a smooth term for the environmental attribute), one set for each cardiometabolic risk factor. GAMMs with Gaussian variance and identity link functions for waist circumference, LDL cholesterol and mean arterial pressure. GAMMs with Gamma variance and logarithmic link function for HDL cholesterol, glycated, triglycerides and glycated haemoglobin.
1Tb*	Street intersection density (intersections/km^2)	Age, sex, English-speaking background, educational attainment, population density, area-level IRSAD	As above
1Tc*	Percentage of commercial land use (% area in residential buffer)	Age, sex, English-speaking background, educational attainment, population density, area-level IRSAD, residential self-selection related to access to destinations	As above
1Td*	Land use mix (entropy score of 5 non-commercial land uses)	Age, sex, English-speaking background, educational attainment, population density, area-level IRSAD, residential self-selection related to access to destinations	As above

1Te*	Percentage of parkland (% of area in residential buffer)	Age, sex, English-speaking background, educational attainment, population density, percentage of commercial land use, land use mix (5 non-commercial land uses), area-level IRSAD, residential self-selection related to recreational facilities	As above
1Tf*	Percentage of blue space (% of area in residential buffer)	Age, sex, English-speaking background, educational attainment, area-level IRSAD, residential self-selection related to recreational facilities, household income	As above
1Tg*	Annual average NO_2 exposure (ppb)	Age, sex, English-speaking background, educational attainment, area-level IRSAD, population density, street intersection density, percentage of commercial land use, land use mix (5 non-commercial land uses), percentage of parkland, residential self-selection related to recreational facilities	As above
1Th*	Annual average $PM_{2.5}$ exposure (μg/m^3)	Age, sex, English-speaking background, educational attainment, area-level IRSAD, population density, street intersection density, percentage of commercial land use, land use mix (5 non-commercial land uses), percentage of parkland, residential self-selection related to recreational facilities	As above
**Estimation of Direct and Mediated Effects of Neighbourhood Environmental Attributes on two Measures of Cognitive Function**			
2Da	Direct effect of population density [exposure 1] on street intersection density, percentage of commercial land and land use mix (5 land uses) [exposures 2]	Age, educational attainment, English-speaking background; residential self-selection related to access to destinations for percentage of commercial land use and land use mix	Three separate GAMMs, one for each environmental attribute (i.e., exposures 3). GAMMs with Gamma variance and logarithmic link functions.
2Db	Direct effect of population density [exposure 1], percentage of commercial land and land use mix (5 non-commercial land uses) [exposures 2] on percentage of parkland [exposure 3]	Age, educational attainment, English-speaking background, residential self-selection related to recreational facilities	A single GAMM with Gamma variance and logarithmic link functions.
2Dc	Direct effect of exposures 1-3 on two measures of air pollution [exposures 4]	Age, educational attainment, English-speaking background, residential self-selection related to recreational facilities	Two separate GAMMs, one for each air pollution measure (i.e., exposures 4). GAMMs with Gaussian variance and identity link function for $PM_{2.5}$ and Gamma variance and logarithmic link function for NO_2.
3Da	Direct effects of exposures 1-4 on waist circumference [cardiometabolic risk factor 1]	Age, educational attainment, sex, English-speaking background, household income, area-level IRSAD, living arrangements, work/volunteer status, smoking status, residential self-selection related to access to destinations and recreational facilities	A single GAMM with Gaussian variance and identity link functions.
3Db*	Direct effects of exposures 1-4 and waist circumference [cardiometabolic risk factor 1] on HDL cholesterol [cardiometabolic risk factor 2]	Age, educational attainment, sex, English-speaking background, household income, area-level IRSAD, living arrangements, work/volunteer status, smoking status, residential self-selection related to access to destinations and recreational facilities	A single GAMM with Gamma variance and logarithmic link functions.

3Dc*	Direct effects of exposures 1–4 and waist circumference [cardiometabolic risk factor 1] on LDL cholesterol [cardiometabolic risk factor 3]	Age, educational attainment, sex, English-speaking background, household income, area-level IRSAD, living arrangements, work/volunteer status, smoking status, residential self-selection related to access to destinations and recreational facilities	A single GAMM with Gaussian variance and identity link functions.
3Dd*	Direct effects of exposures 1–4 and waist circumference [cardiometabolic risk factor 1] on triglycerides [cardiometabolic risk factor 4]	Age, educational attainment, sex, English-speaking background, household income, area-level IRSAD, living arrangements, work/volunteer status, smoking status, residential self-selection related to access to destinations and recreational facilities	A single GAMM with Gamma variance and logarithmic link functions.
3De*	Direct effects of exposures 1–4 and waist circumference [cardiometabolic risk factor 1] on glycated haemoglobin [cardiometabolic risk factor 5]	Age, educational attainment, sex, English-speaking background, household income, area-level IRSAD, living arrangements, work/volunteer status, smoking status, residential self-selection related to access to destinations and recreational facilities	A single GAMM with Gamma variance and logarithmic link functions.
3Df*	Direct effects of exposures 1-4 and waist circumference [cardiometabolic risk factor 1] on mean arterial pressure [cardiometabolic risk factor 6]	Age, educational attainment, sex, English-speaking background, household income, area-level IRSAD, living arrangements, work/volunteer status, smoking status, residential self-selection related to access to destinations and recreational facilities	A single GAMM with Gaussian variance and identity link functions.
4D*[1]	Direct effects of exposures 1–4 and cardiometabolic risk factors 1–6 on two measures of cognitive function (memory and processing speed) [outcomes]	Age, sex, educational attainment, English-speaking background, household income, living arrangements, work/volunteer status, smoking status, history of heart problems / stroke, residential self-selection related to access to destinations and recreational facilities, area-level IRSAD	Two separate sets of GAMMs (one GAMM with a linear and another with a smooth term for the environmental attribute), one set for each cognitive outcome. GAMMs with Gaussian variance and identity link functions for CVLT and SDMT.

Note. IRSAD, Index of Relative Social Advantage and Disadvantage; GAMM, generalised additive mixed model; HDL, high-density lipoprotein; LDL, low-density lipoprotein; "T" in the Step column refers to "total effect", while "D" refers to "direct effect". * The moderating effects of medications for a specific cardiometabolic risk factor on environment-cardiometabolic risk factor associations were examined by adding two-way interaction terms.*[1] The moderating effect of medications for a specific cardiometabolic risk factor on environment-cognition and cardiometabolic risk factor-cognition associations and were examined by adding two-way interaction terms.

Section S3. Supplementary results

Table S2 reports the results of the GAMMs estimating the relationships between neighbourhood environmental characteristics. Figures S2 to S4 depict the curvilinear relationships mentioned in Table S2.

Table S2. Relationships between neighbourhood environmental variables.

Models	Direct Effect of …	…on (Response Variable)	Statistic	Statistic Values	*p*-Value
2Da.1	Population density (person/ha)	**Street intersection density** (intersections/km^2)	*F*-ratio (df1, df2)	341.82 (8.72, 4131.28) see Figure S2A	**<.001**
2Da.2		**Percentage of commercial land**	*F*-ratio (df1, df2)	24.71 (6.37, 4132.63) see Figure S2B	**<.001**
2Da.3		**Non-commercial land use mix**	*F*-ratio (df1, df2)	10.53 (4.85, 4134.15) see Figure S2C	**<.001**

2Db	Population density (person/ha)	**Percentage of parkland**	*F*-ratio (df1, df2)	13.88 (2.98, 4134.02) see Figure S2D	**<.001**
	Percentage of commercial land		e^b (95% CI)	0.997 (0.992, 1.003)	.306
	Non-commercial land use mix (entropy score)		e^b (95% CI)	0.691 (0.538, 0.888)	**.004**
2Dc.1	Population density (person/ha)	**NO_2** (ppb)	*F*-ratio (df1, df2)	72.83 (7.05, 4124.05) see Figure S3A	**<.001**
	Street intersection density (intersections/km^2)		*F*-ratio (df1, df2)	11.45 (1.98, 4124.05) see Figure S5 (left panel)	**<.001**
	Non-commercial land use mix (entropy score)		*F*-ratio (df1, df2)	44.76 (2.93, 4124.05) see Figure S5 (right panel)	**<.001**
	Percentage of commercial land		e^b (95% CI)	1.006 (1.005, 1.007)	**<.001**
	Percentage of parkland		e^b (95% CI)	1.0010 (1.0005, 1.0015)	**<.001**
2Dc.2	Population density (person/ha)	**$PM_{2.5}$** (μg/m^3)	*F*-ratio (df1, df2)	9.94 (6.88, 4127.12) see Figure S3B	**<.001**
	Street intersection density (intersections/km^2)		*b* (95% CI)	-0.0001 (-0.0006, 0.0004)	.718
	Non-commercial land use mix (entropy score)		*b* (95% CI)	0.093 (0.030, 0.153)	**.004**
	Percentage of commercial land		*b* (95% CI)	0.004 (0.003, 0.006)	**<.001**
	Percentage of parkland		*b* (95% CI)	0.002 (0.001, 0.002)	**<.001**

Notes*:* F-ratio, F-ratio for smooth term defining a curvilinear relationship; df = degrees of freedom; *b*, regression coefficient; CI, confidence interval; e^b, exponentiated regression coefficient (from GAMMs with Gamma variance and logarithmic link function); in bold are effects significant at the probability level of 0.05.

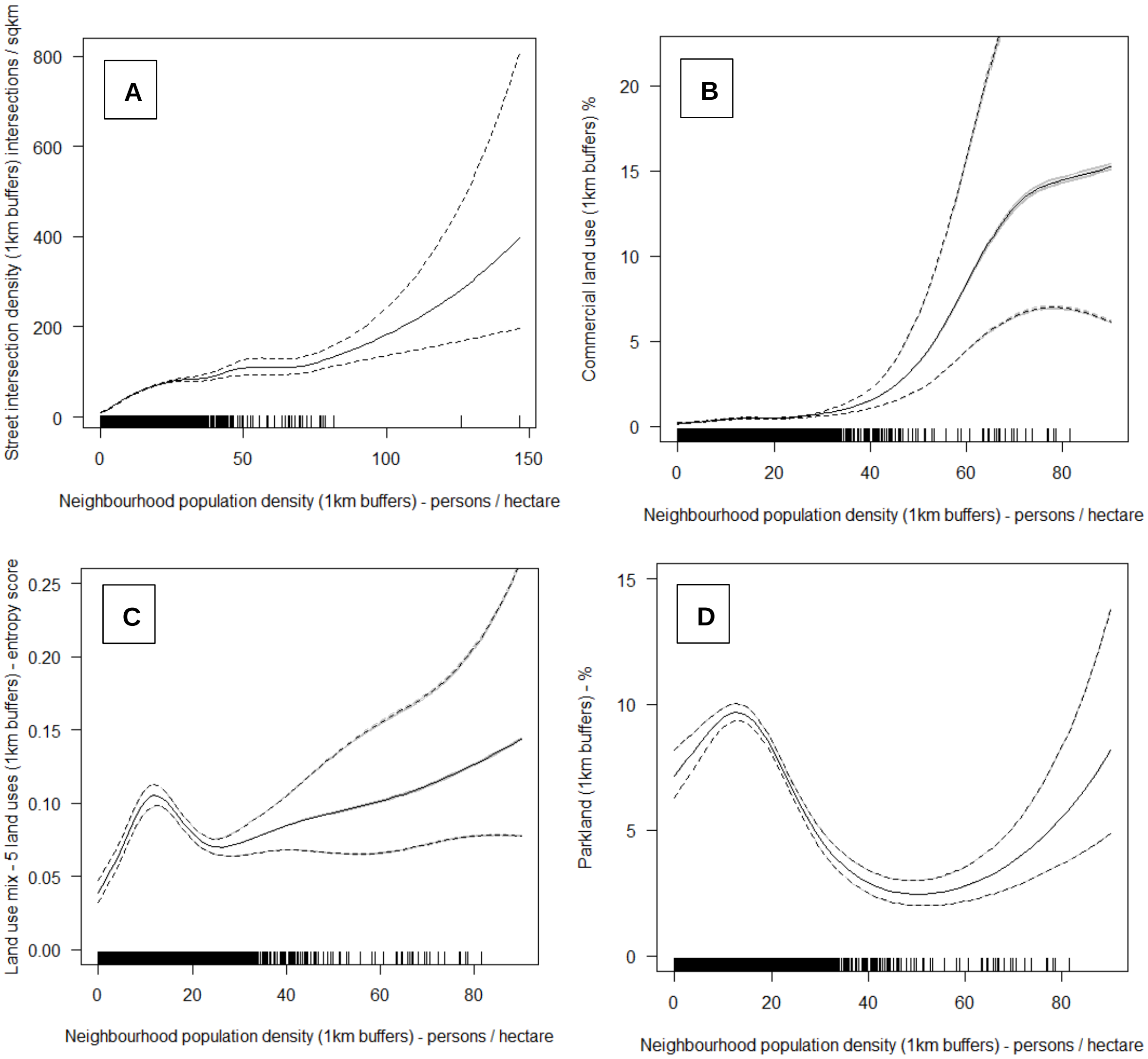

Figure S2. Curvilinear relationships of population density with street intersection density **(A),** percentage of commercial land use (**B**), non-commercial land use mix **(C)** and percentage parkland **(D)** in 1km residential buffers.

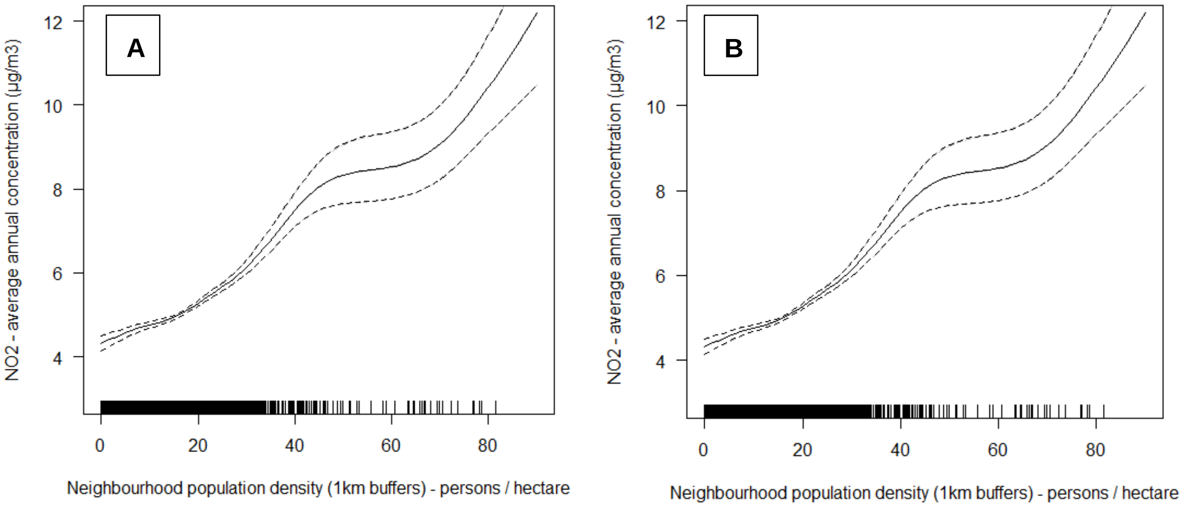

Figure S3. Curvilinear relationships of population density with average annual concentrations of NO_2 (**A**) and $PM_{2.5}$ (**B**).

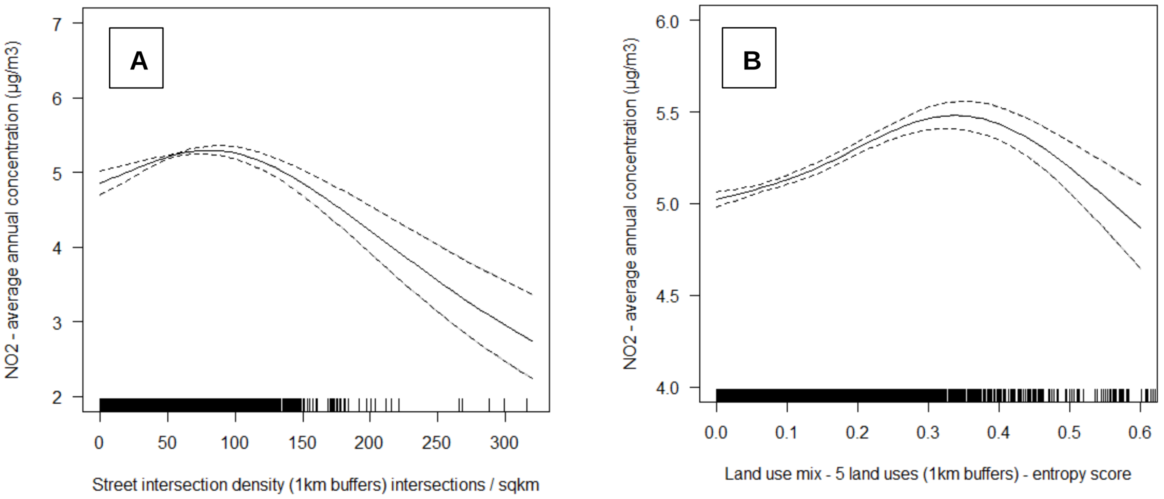

Figure S4. Curvilinear relationships of street intersection density **(A)** and non-commercial land use mix **(B)** with average annual concentrations of NO_2.

Table S3 shows the results of the GAMMs estimating the direct effects of neighbourhood environmental characteristics on cardiometabolic risk factors. Here, the results are presented in the original units of the environmental variables, while the results presented in the main manuscript have been rescaled (e.g., a unit of 10 persons/ha rather than 1 person/ha for population density) to avoid reporting small values for the regression coefficients.

Table S3. Relationships between neighbourhood environmental characteristics and cardiometabolic risk factors – direct effects.

Models	Direct effect of …	… on (response variable)	Statistic	Statistic values	*p*-value
3Da	Population density (person/ha)	**Waist circumference** (cm)	*b* (95% CI)	-0.044 (-0.112, 0.024)	.206
	Street intersection density (intersections/km^2)		*b* (95% CI)	0.003 (-0.015, 0.020)	.752
	Percentage of commercial land		*b* (95% CI)	0.006 (-0.080, 0.067)	.869
	Non-commercial land use mix (entropy score)		*b* (95% CI)	-0.068 (-3.888, 3.752)	.972
	Percentage of parkland		*b* (95% CI)	-0.020 (-0.058, 0.018)	.293
	Percentage of blue space		*b* (95% CI)	-0.322 (-0.526, -0.118)	**.002**
	NO_2 (ppb)		*b* (95% CI)	0.037 (-0.265, 0.339)	.811
	$PM_{2.5}$ (µg/m^3)		*b* (95% CI)	0.347 (0.047, 0.646)	**.024**
3Db	Population density (person/ha)	**HDL cholesterol** (mg/dL)	e^b (95% CI)	0.9998 (0.9985, 1.0010)	.713
	Street intersection density (intersections/km^2)		e^b (95% CI)	1.0003 (1.0000, 1.0006)	**.048**
	Percentage of commercial land		e^b (95% CI)	0.9991 (0.9978, 1.0005)	.200
	Non-commercial land use mix (entropy score)		e^b (95% CI)	0.953 (0.888, 1.023)	.183
	Percentage of parkland		e^b (95% CI)	0.9994 (0.9987, 1.0001)	.094
	Percentage of blue space		e^b (95% CI)	1.001 (0.998, 1.005)	.446
	NO_2 (ppb)		e^b (95% CI)	0.999 (0.993, 1.004)	.675
	$PM_{2.5}$ (µg/m^3)		e^b (95% CI)	0.993 (0.986, 0.998)	**.030**
	Waist circumference (cm)		e^b (95% CI)	0.994 (0.993, 0.995)	**<.001**
3Dc	Population density (person/ha)	**LDL cholesterol** (mg/dL)	*b* (95% CI)	-0.003 (-0.007, 0.001)	.130
	Street intersection density (intersections/km^2)		*b* (95% CI)	0.001 (0.00002, 0.002)	**.045**
	Percentage of commercial land		*b* (95% CI)	-0.002 (-0.006, 0.002)	.329
	Non-commercial land use mix (entropy score)		*b* (95% CI)	0.139 (-0.074, 0.352)	.200
	Percentage of parkland		*b* (95% CI)	-0.002 (-0.004, -0.0001)	**.046**

	Percentage of blue space		*b* (95% CI)	-0.006 (-0.018, 0.006)	.319
	NO_2 (ppb)		*b* (95% CI)	-0.002 (-0.019, 0.014)	.780
	$PM_{2.5}$ (μg/m³)		*b* (95% CI)	0.0159 (0.0001, 0.0319)	**.049**
	Waist circumference (cm)		*b* (95% CI)	0.002 (0.001, 0.004)	**.009**
3Dd	Population density (person/ha)	**Triglycerides** (mg/dL)	e^b (95% CI)	0.998 (0.996, 1.001)	.313
	Street intersection density (intersections/km²)		e^b (95% CI)	1.0007 (1.0001, 1.0014)	**.039**
	Percentage of commercial land		e^b (95% CI)	1.001 (0.999, 1.004)	.331
	Non-commercial land use mix (entropy score)		e^b (95% CI)	1.057 (0.920, 1.219)	.428
	Percentage of parkland		e^b (95% CI)	1.001 (0.999, 1.002)	.365
	Percentage of blue space		e^b (95% CI)	1.003 (0.994, 1.011)	.606
	NO_2 (ppb)		e^b (95% CI)	0.998 (0.987, 1.009)	.684
	$PM_{2.5}$ (μg/m³)		e^b (95% CI)	1.006 (0.995, 1.017)	.324
	Waist circumference (cm)		e^b (95% CI)	1.012 (1.010, 1.013)	**<.001**
3De	Population density (person/ha)	**Glycated haemoglobin** (mmol/mol)	e^b (95% CI)	1.001 (0.999, 1.002)	.194
	Street intersection density (intersections/km²)		e^b (95% CI)	1.000 (0.9998, 1.0002)	.738
	Percentage of commercial land		e^b (95% CI)	0.999 (0.998, 1.001)	.202
	1. Non-commercial land use mix (entropy score)		e^b (95% CI)	0.955 (0.887, 1.028)	.215
	Percentage of parkland		e^b (95% CI)	1.000 (0.997, 1.0004)	.801
	Percentage of blue space: *in those without diabetes medication*		e^b (95% CI)	0.9998 (0.998, 1.002)	.840
	Percentage of blue space: *in those with diabetes medication*		e^b (95% CI)	0.985 (0.973, 0.998)	**<.001**
	NO_2 (ppb)		e^b (95% CI)	1.004 (1.001, 1.006)	**.007**
	$PM_{2.5}$ (μg/m³)		e^b (95% CI)	1.000 (0.997, 1.003)	.964
	Waist circumference (cm)		e^b (95% CI)	1.0018 (1.0016, 1.0021)	**<.001**
3Df	Population density (person/ha)	**Mean arterial blood pressure** (mmHg)	*b* (95% CI)	-0.125 (-0.183, -0.066)	**<.001**
	Street intersection density (intersections/km²)		*b* (95% CI)	0.055 (0.040, 0.070)	**<.001**
	Percentage of commercial land		*b* (95% CI)	0.045 (-0.018, 0.109)	.160
	Non-commercial land use mix (entropy score)		*b* (95% CI)	4.532 (1.247, 7.819)	**.007**
	Percentage of parkland		*b* (95% CI)	-0.007 (-0.040, 0.026)	.671
	Percentage of blue space		*b* (95% CI)	-0.059 (-0.237, 0.119)	.516
	NO_2 (ppb)		*b* (95% CI)	-0.397 (-0.655, -0.139)	**.003**
	$PM_{2.5}$ (μg/m³)		*b* (95% CI)	0.255 (0.002, 0.513)	**.048**
	Waist circumference (cm)		*b* (95% CI)	0.209 (0.182, 0.237)	**<.001**

Note. *b*, regression coefficient; CI, confidence interval; e^b, exponentiated regression coefficient (from GAMMs with Gamma variance and logarithmic link function); in bold are effects significant at the probability level of 0.05.

References

1. Adams MA, Frank LD, Schipperijn J; et al. International variation in neighborhood walkability, transit, and recreation environments using geographic information systems: The IPEN adult study. *Int. J. Health Geogr*. **2014**, *13*, 43. https://doi.org/10.1186/1476-072X-13-43.
2. Cerin, E.; Van Dyck, D.; Zhang, C.J.; Van Cauwenberg, J.; Lai, P.C.; Barnett, A. Urban environments and objectively-assessed physical activity and sedentary time in older Belgian and Chinese community dwellers: Potential pathways of influence and the moderating role of physical function. *Int. J. Behav. Nutr. Phys. Act*. **2020**, *17*, 73. https://doi.org/10.1186/s12966-020-00979-8.
3. Cochrane, T.; Yu, Y.; Davey, R.; Cerin, E.; Cain, K.L.; Conway, T.L.; Kerr, J.; Frank, L.D.; Chapman, J.E.; Adams, M.A.; et al. Associations of built environment and proximity of food outlets with weight status: Analysis from 14 cities in 10 countries. *Prev. Med*. **2019**, *129*, 105874. https://doi.org/10.1016/j.ypmed.2019.105874.
4. Cerin, E.; Conway, T.L.; Cain, K.L.; Kerr, J.; De Bourdeaudhuij, I.; Owen, N.; Reis, R.S.; Sarmiento, O.L.; Hinckson, E.A.; Salvo, D.; et al. Sharing good NEWS across the world: Developing comparable scores across 12 countries for the Neighborhood Environment Walkability Scale (NEWS). *BMC Public Health* **2013**, *13*, 309. https://doi.org/10.1186/1471-2458-13-309.
5. Gunn, L.D.; King, T.L.; Mavoa, S.; Lamb, K.E.; Giles-Corti, B.; Kavanagh, A. Identifying destination distances that support walking trips in local neighbourhoods. *J. Trans. Health* **2017**, *5*, 133–149. https://doi.org/10.1016/j.jth.2016.08.009.

6. ABS. *Census of Population and Housing: Mesh Block Counts, 2011 (Cat. No. 2074)*; Australian Bureau of Statistics: Canberra, Australia, 2011.
7. Cerin, E.; Barnett, A.; Zhang, C.J.; Lai, P.C.; Sit, C.H.; Lee, R.S. How urban densification shapes walking behaviours in older community dwellers: A cross-sectional analysis of potential pathways of influence. *Int. J. Health Geogr*. **2020**, *19*, 14. https://doi.org/10.1186/s12942-020-00210-8.
8. Sallis, J.F.; Cerin, E.; Kerr, J.; Adams, M.A.; Sugiyama, T.; Christiansen, L.B.; Schipperijn, J.; Davey, R.; Salvo, D.; Frank, L.D.; et al. Built environment, physical activity, and obesity: Findings from the International Physical Activity and Environment Network (IPEN) Adult Study. *Annu. Rev. Public Health* **2020**, *41*, 119–139. https://doi.org/10.1146/annurev-publhealth-040218-043657.
9. Cerin, E; Nathan, A.; Van Cauwenberg, J.; Barnett, D.W.; Barnett, A. The neighbourhood physical environment and active travel in older adults: A systematic review and meta-analysis. *Int. J. Behav. Nutr. Phys. Act*. **2017**, *14*, 15. https://doi.org/10.1186/s12966-017-0471-5.
10. Hand, C.L.; Howrey, B.T. Associations among neighborhood characteristics, mobility limitation, and social participation in late life. *J. Gerontol. B Psychol. Sci. Soc. Sci*. **2019**, *74*, 546–555. https://doi.org/10.1093/geronb/gbw215.
11. PSMA Australia Ltd. PSMA street network; 2012.
12. Zhang, C.J.; Barnett, A.; Johnston, J.M.; Lai, P.C.; Lee, R.S.; Sit, C.H.; Cerin, E. Objectively-measured neighbourhood attributes as correlates and moderators of quality of life in older adults with different living arrangements: The ALECS cross-sectional study. *Int. J. Environ. Res. Public Health* **2019**, *16*, 876. https://doi.org/10.3390/ijerph16050876.
13. ABS. *Australian Statistical Geography Standard (ASGS) Volume 1—Main Structure and Greater Capital City Statistical Areas (Cat No. 1270.0.55.001)*; Australian Bureau of Statistics: Canberra, Australia, 2011.
14. Frank, L.D.; Sallis, J.F.; Saelens, B.E.; Leary, L.; Cain, K.; Conway, T.L.; Hess, P.M. development of a walkability index: Application to the Neighborhood Quality of Life Study. *Br. J. Sports Med*. **2010**, *44*, 924–933.
15. Besser, L.M.; McDonald, N.C.; Song, Y.; Kukull, W.A.; Rodriguez, D.A. Neighborhood environment and cognition in older adults: A systematic review. *Am. J. Prev. Med.* **2017**, *53*, 241–251. https://doi.org/10.1016/j.amepre.2017.02.013.
16. Crossman, S.; Li, O. *Surface Hydrology Polygons (National)*; Geoscience Australia: Canberra, Australia, 2015.
17. Barton, J.; Rogerson, M. The importance of greenspace for mental health. *BJPsych Int*. **2017**, *14*, 79–81. https://doi.org/10.1192/s2056474000002051.
18. Maas, J.; van Dillen, S.M.E.; Verheij, R.A.; Groenwegen, P.P. Social contacts as a possible mechanism behind the relation between green space and health. *Health Place* **2009**, *15*, 586–595. https://doi.org/10.1016/j.healthplace.2008.09.006.
19. de Keijzer, C.; Gascon, M.; Nieuwenhuijsen, M.J.; Dadvand, P. Long-term green space exposure and cognition across the life course: A systematic review. *Curr. Environ. Health Rep*. **2016**, *3*, 468–477. https://doi.org/10.1007/s40572-016-0116-x.
20. Hirabayashi, S.; Nowak, D.J. Comprehensive national database of tree effects on air quality and human health in the United States. *Environ. Pollut*. **2016**, *215*, 48–57. https://doi.org/10.1016/j.envpol.2016.04.068.
21. Kummu, M.; De Moel, H.; Ward, P.J.; Varis, O. How close do we live to water? A global analysis of population distance to freshwater bodies. *PLoS ONE* **2011**, *6*, e20578. https://doi.org/10.1371/journal.pone.0020578.
22. Tundi, A.; Alder, J. Coastal Systems. In *Rashin Hassan, Robert Scholes, and Neville Ash, eds, Ecosystems and Human Well-Being: Current State and Trends*; Island Press: Washington, DC, USA, 2005; Volume 1.
23. Pasanen, T.P.; White, M.P.; Wheeler, B.W.; Garrett, J.K.; Elliott, L.R. Neighbourhood blue space, health and wellbeing: The mediating role of different types of physical activity. *Environ. Int*. **2019**, *131*, 105016. https://doi.org/10.1016/j.envint.2019.105016.
24. Volker, S.; Kistemann, T. Developing the urban blue: Comparative health responses to blue and green urban spaces in Germany. *Health Place* **2011**, *35*, 196–205.
25. Gascon, M.; Triguero-Mas, M.; Martínez, D.; Dadvand, P.; Forns, J.; Plasència, A.; Nieuwenhuijsen, M.J. Mental health benefits of long-term exposure to residential green and blue spaces: A systematic review. *Int. J. Environ. Res. Public Health*. **2015**, *12*, 4354–4379.
26. Rubin D.B. Multiple imputation for non–response in surveys. *Wiley J & Sons*, New York USA, **1987**, *14* No.11. http://dx.doi.org/10.1002/9780470316696
27. Van Buuren S. Flexible imputation of missing data, 2nd ed. *Chapman & Hall*, Boca Raton, Florida, USA, **2018.**
28. van Buuren S., Groothuis-Oudshoorn K. mice: Multivariate Imputation by Chained Equations in R. *J Stat Softw*. **2011**;*45* (3):1–67.
29. R. Core Team. R.: A language and environment for statistical computing. *R Foundation for Statistical Computing*, Vienna, Austria. **2020**. https://www.R-project.org/.
30. Wood S.N. Generalized additive models: an introduction with R, 2nd ed. Chapman & Hall/CRC, Boca Raton, Florida, USA, **2017.**
31. Sheather S. A modern approach to regression with R. Springer, New York, USA **2009.**
32. MacKinnon D.P.; Luecken L.J. How and for whom? Mediation and moderation in health psychology. *Health Psychol*. **2008**;*27* (2S):S99–S100. doi: 10.1037/0278–6133.27.2(Suppl.)
33. Burnham K.P.; Anderson D.R.. Model selection and multimodel inference: A practical information-theoretic approach, 2nd ed. Springer Verlag, New York, USA, **2002.**
34. Cerin E.; Conway T.L.; Adams M.A.; Barnett A.; Cain K.L.; Owen N.; Christiansen L.B.; Dyck D.V., Mitáš J.; Sarmiento O. L. et al. Objectively-assessed neighbourhood destination accessibility and physical activity in adults from 10 countries: An analysis of moderators and perceptions as mediators. *Soc Sci. Med.* **2018**, *211* :282–293. doi: 10.1016/j.socscimed.2018.06.034

35. Cerin E.; Barnett A.; Chaix B.; Chaix B.; Nieuwenhuijsen M.J.; Caeyenberghs K.; Jalaludin B.; Sugiyama T.; Sallis J.F.; Lautenschlager N.T. et al. International Mind, Activities and Urban Places (iMAP) study: methods of a cohort study on environmental and lifestyle influences on brain and cognitive health. *BMJ Open*. **2020**, 10 (3):e036607. doi: 10.1136/bmjopen-2019-036607
36. Anstey K.J.; Sargent-Cox K.; Eramudugolla R. et al. Association of cognitive function with glucose tolerance and trajectories of glucose tolerance over 12 years in the AusDiab study. *Alzheimers Res. Ther*. **2015**, 7 (1):48. doi: 10.1186/s13195-015-0131-4
37. Bays H.E.; Toth P.P.; Kris-Etherton P.M.; Abate N; Aronne L.J.; Brown W.V.; Gonzalez-Campoy J.M.; Jones S.R.; Kumar R.; La Forge R.; Samuel V.T.; Obesity, adiposity, and dyslipidemia: a consensus statement from the National Lipid Association. *J. Clin. Lipidol*. **2013**, *7* (4):304–383. doi: 10.1016/j.jacl.2013.04.001.
38. Klein S.; Allison D.B.; Heymsfield S.B.; Kelley D.E.; Leibel R.L.; Nonas C; Kahn R. Waist circumference and cardiometabolic risk: a consensus statement from shaping America's health: Association for Weight Management and Obesity Prevention; NAASO, the Obesity Society; the American Society for Nutrition; and the American Diabetes Association. *Obesity*. **2007**, 15 (5):1061–1067. doi: 10.1038/oby.2007.632.
